# Supplementary material for: FedEmoNet: Privacy-preserving federated learning with TCN-Transformer fusion for cross-corpus speech emotion recognition
Source: PLoS One. 2026 May 7;21(5):e0342953. doi: 10.1371/journal.pone.0342953 (PMC13152135; doi:10.1371/journal.pone.0342953)
Supplement: Attachment — Submitted filename: response_to_reviewers.pdf [file pone.0342953.s001.pdf]

Response to Reviewers

Manuscript ID: PONE-D-26-04975

Multi-Scale Federated Learning with Differential Privacy: A TCN-Transformer Framework for Cross-Corpus Speech Emotion Recognition

PLOS ONE

**Corresponding Author:**	Mohammed Tawfik
**Email:**	M.tawfik@su.edu.ye
**ORCID:**	0000-0002-1227-387X
**Affiliations:**	Faculty of Computer and Information Technology, Sana'a University, Yemen<br>Department of Computer Science, Ajloun National University, Jordan

Dear Dr. Viacheslav Kovtun (Academic Editor) and Reviewers,

We sincerely thank the Academic Editor and all three reviewers for their thorough and constructive evaluation. We have carefully addressed every point, substantially revising the manuscript. The abstract has been rewritten for clarity and stronger gap identification. All figures have been regenerated at 300 DPI and are uploaded separately per PLOS guidelines. Below we provide point-by-point responses.

Journal Requirements

Requirement 1 — PLOS ONE style and LaTeX template

Please ensure that your manuscript meets PLOS ONE's style requirements. Please update your submission to use the PLOS LaTeX template.

The manuscript has been completely reformatted using the official PLOS ONE LaTeX template (Version 3.7, August 2025) with `plos2025.bst`. Title follows sentence case. Author names, affiliations, and corresponding author email follow the prescribed format. Figures contain captions only (no embedded graphics) per PLOS guidelines. Tables use cell-based formatting without nested tabular environments. References follow Vancouver style.

Requirement 2 — Code sharing

Please review our guidelines and ensure that your code is shared in a way that follows best practice.

All author-generated code underpinning the findings—including the federated training framework, TCN-Transformer model, PSO feature selection pipeline, and explainability analysis scripts—will be made available upon reasonable request to the corresponding author. This is stated in the manuscript.

See Data Availability Statement in the revised manuscript.

Requirement 4 — Data repository

Please upload the minimal data set necessary to replicate your study's findings to a stable, public repository.

All three datasets used in this study are publicly available from their original repositories. The Data Availability Statement now provides direct URLs: EmoDB (`http://emodb.bilderbar.info/`), RAVDESS (`https://zenodo.org/record/1188976`), and CREMA-D (`https://github.com/CheyneyComputerScience/CREMA-D`). No custom dataset was created; all experiments are fully reproducible using these public sources.

Requirement 5 — ORCID iD

Please ensure that you have an ORCID iD and that it is validated in Editorial Manager.

The corresponding author's ORCID iD (0000-0002-1227-387X) has been verified and validated in Editorial Manager.

Requirement 6 — Figures 9 and 12 clarity

Please upload a new copy of Figures 9 and 12 as the details are not clear.

All figures have been regenerated at 300 DPI with larger fonts, clearer labels, and improved color contrast. In particular, confusion matrices now display all numerical values prominently (new Fig 6). The CREMA-D per-emotion breakdown is presented as a dedicated new figure (Fig 7). All figures are uploaded separately as required.

Requirement 7 — Reference Tables 1 and 3 in text

Please ensure that you refer to Tables 1 and 3 in your text.

All tables are now explicitly referenced in the text at first mention. Table 1 (Related Work Summary) is cited in the opening sentence of the Related Work section. Table 3 (PSO feature selection) is cited in the PSO-Optimized Feature Selection subsection.

Reviewer #1

Comment 1 — Cross-corpus generalization analysis

(a–b) The 68.15% accuracy on CREMA-D requires deeper analysis. Provide per-emotion breakdown for CREMA-D.

We have added:

- New Table 9 with per-emotion Precision, Recall, F1-Score, and Support for all six CREMA-D emotions, annotated by arousal level
- New Fig 7 with (a) grouped bar chart of per-emotion metrics and (b) arousal-based transfer analysis showing high-arousal at 71.9% vs. low-arousal at 62.1%
- Clear numerical confusion matrix for CREMA-D in Fig 6(c)

See Section "Cross-corpus generalization on CREMA-D," Table 9, Fig 6(c), and Fig 7.

(c) Analyze why neutral performs poorly (58%).

Three factors are identified: (1) Cultural and linguistic variation in neutral expression between German (EmoDB), North American English (RAVDESS), and crowd-sourced English (CREMA-D); (2) Greater speaker diversity in CREMA-D (91 actors vs. 10 in EmoDB); (3) Annotation methodology differences—crowd-sourced labeling introduces noise for ambiguous low-arousal categories.

See "Analysis of the cross-corpus performance gap" in the Results section.

(d) Discuss whether the 30% gap fundamentally limits the "cross-corpus" claim.

The 68.15% represents meaningful zero-shot generalization across two languages and three recording conditions. High-arousal emotions achieve 71.9%, confirming acoustic universals in f_0 and energy. This result matches the 68% reported by FedSER-XAI [9] on the same CREMA-D benchmark. The gap is primarily driven by low-arousal categories—particularly neutral—rather than a fundamental framework limitation.

(e) Consider adding t-SNE visualization of feature distributions across datasets.

New Fig 9 provides a three-panel t-SNE visualization: (a) dataset-colored view showing domain shift; (b) emotion-colored view revealing cross-dataset clustering for high-arousal emotions; (c) domain shift highlighting CREMA-D relative to training data.

See new Fig 9 and accompanying discussion.

Comment 2 — Data leakage verification

(a) Confirm no speakers appear in both training and test sets.

Explicitly confirmed: the global 80/20 split is performed before federated distribution. RAVDESS uses subject-independent splitting; EmoDB uses actor-level stratification. Both guarantee complete speaker disjointness.

(b) Details on the “mixed client” composition.

Now specified: Client 5 contains 30% EmoDB (∼130 samples) and 70% RAVDESS (∼300 samples), testing cross-lingual heterogeneity.

(c) Whether preprocessing was applied globally or per-client.

Added: all preprocessing (VAD, normalization, resampling) is applied per-client independently after distribution, preventing cross-client leakage through global statistics.

See “Data leakage prevention” paragraph in the Methodology section.

Comment 3 — Near-perfect performance scrutiny

(a) Report confusion matrix values numerically.

New Fig 6 provides clear numerical confusion matrices for all three datasets with large, readable annotations at 300 DPI.

(b) Perform ablation with reduced training data to test memorization.

New Fig 10 shows monotonic degradation: 88.79% (20%), 93.46% (40%), 96.26% (60%), 98.13% (80%), 99.07% (100%). This rules out memorization—a memorizing model would maintain high accuracy until critical samples were removed, then drop sharply.

See new “Near-perfect performance validation” subsection and Fig 10.

(c) Compare against human-level performance.

Added: EmoDB human recognition ∼84–86% [35]; RAVDESS ∼60–72% [34]. Exceeding human performance is consistent with deep learning advances on acted speech where acoustic patterns are stereotypical.

(d) Discuss ceiling effects.

EmoDB (535 samples, acted speech) has well-documented ceiling effects with multiple methods >95%. CREMA-D (7,442 samples, 91 actors) is the more meaningful benchmark where 68.15% is well below ceiling.

Comment 4 — PSO optimization details

(a) How was the fitness function computed?

Fitness uses 5-fold cross-validation on each client's local data with a lightweight proxy classifier (single hidden layer, 256 units, 10 epochs).

(b) Discuss computational cost of 1,000 model trainings.

Using the proxy classifier ($\sim$2.7 sec/evaluation), total PSO time is $\sim$45 min/client—modest compared to 5.2 h total training. Breakdown in Fig 8(c) and Table 14.

(c) Report convergence behavior.

New Fig 8 shows: (a) fitness convergence stabilizing by iteration 35; (b) feature count reduction 150→103; (c) cost breakdown.

Comments 5–8 — Writing improvements

Reduce redundancy, consolidate sections, fix figure ordering, deeper cross-corpus analysis.

(1) Abstract rewritten to eliminate redundancy with Introduction and strengthen gap identification; (2) Methodology consolidated into cleaner subsections; (3) All figures referenced sequentially; (4) Cross-corpus analysis substantially expanded as detailed above.

Reviewer #2

Major Revision 1 — Multi-scale PSR parameter selection

Elaborate on the multi-scale phase space reconstruction methodology and parameter selection rationale.

Expanded with: (1) $\tau = 17$ justified via AMI analysis following Fraser & Swinney (1986); (2) $d = 3$ justified via FNN algorithm; (3) psychoacoustic motivation for the three scales—micro aligns with critical band window, meso with syllabic rate (4–8 Hz), macro with prosodic phrase duration.

See "Multi-scale phase space reconstruction" in Methodology.

Major Revision 2 — Federated client setup

Provide more details on the federated client setup and non-IID data generation process.

Now includes: explicit per-client sample counts, mixed client 30/70 ratio, Dirichlet sampling parameters, and per-client preprocessing procedures.

Major Revision 3 — Additional datasets

Include additional diverse datasets.

Adding entirely new datasets would require significant additional experimentation beyond the revision scope. We have instead: (1) strengthened CREMA-D evaluation with per-emotion analysis, t-SNE visualization, and arousal-based transfer analysis; (2) discussed comparability with FedSER-XAI results; (3) identified evaluation on additional languages and naturalistic speech as future work.

Major Revision 4 — Deployment challenges

Discuss practical deployment challenges and potential solutions.

New "Practical deployment considerations" subsection covers: communication overhead and compression strategies, client heterogeneity and asynchronous aggregation, language diversity limitations, and acted vs. naturalistic speech challenges.

Minor Revisions 1–4

Computational efficiency, privacy analysis, statistical validation, consistent notation.

(1) Efficiency table expanded with PSO time; (2) Privacy analysis extended with gradient inversion and model inversion attack evaluation; (3) Statistical validation retained with Bonferroni-corrected t-tests and supplemented by reduced data ablation; (4) Notation made consistent throughout (scalars italic, vectors bold, matrices bold uppercase).

Reviewer #3

Comment 1 — Clarify conclusions with critical analysis

Clarify the conclusions better, discuss the results, and support them with critical analysis.

The Conclusion has been substantially revised with specific numerical findings, per-emotion cross-corpus summary, discussion of limitations (ceiling effects, neutral variability, acted speech), and concrete future work directions.

Comment 2 — Research gap in abstract

Clarifying the research gap in the abstract and strengthening it with the datasets used.

The abstract has been completely rewritten. It now opens with the research gap ("Current federated learning approaches for SER remain limited...lacking formal privacy guarantees"), names all three datasets, and includes the per-emotion arousal-based analysis as a key finding.

Comment 3 — Related work comparison table

A table should be provided summarizing the relevant works in terms of their strengths and weaknesses.

New Table 1 compares eight key methods across: Key Approach, Strengths, Limitations, and FedEmoNet Advances. This clearly maps how our framework addresses each identified gap.

See Table 1 in the Related Work section.

Comment 4 — XAI methods comparison

Explaining the difference between the XAI methods presented.

Added: (1) New Table 5 comparing LIME and SHAP across scope, theoretical basis, faithfulness, cost, and role in FedEmoNet; (2) New Fig 5 directly comparing SHAP and LIME attributions for the same sample ($r = 0.997$ agreement); (3) Expanded text explaining when and why each method is used.

See Table 5, Fig 5, and "Explainable AI framework" subsection.

Additional Citation

Following the evaluation of relevant prior work, we have incorporated the FedSER-XAI paper (Alkhamali et al., Scientific Reports, 2025; DOI: 10.1038/s41598-025-28686-z) throughout the manuscript:

- **Introduction:** Discussed as context for federated XAI in SER, with explicit comparison of capabilities
- **Related Work:** Included in both the FL subsection text and the comparison Table 1
- **State-of-the-art comparison:** Added to Table 13 with a footnote distinguishing centralized vs. federated performance
- **Cross-corpus discussion:** Referenced for benchmarking the 68% CREMA-D result

Summary of New/Revised Elements

**Element**	**Type**	**Addresses**
Table 1: Related work comparison	New	Reviewer 3, Comment 3
Table 5: LIME vs SHAP comparison	New	Reviewer 3, Comment 4
Table 9: CREMA-D per-emotion metrics	New	Reviewer 1, Comment 1b
Fig 5: XAI methods comparison	New (300 DPI)	Reviewer 3, Comment 4
Fig 6: Numerical confusion matrices	New (300 DPI)	Reviewer 1, Comment 3a
Fig 7: CREMA-D per-emotion breakdown	New (300 DPI)	Reviewer 1, Comment 1b
Fig 8: PSO convergence	New (300 DPI)	Reviewer 1, Comment 4c
Fig 9: t-SNE domain shift	New (300 DPI)	Reviewer 1, Comment 1e
Fig 10: Reduced data ablation	New (300 DPI)	Reviewer 1, Comment 3b
Abstract	Rewritten	Reviewer 3, Comment 2
PLOS LaTeX template	Reformatted	Journal Req. 1, 3
